# Identifying diabetic patients with cardiac autonomic neuropathy by heart rate complexity analysis

**DOI:** 10.1186/1475-925X-8-3

**Published:** 2009-01-29

**Authors:** Ahsan H Khandoker, Herbert F Jelinek, Marimuthu Palaniswami

**Affiliations:** 1 Dept. of Electrical & Electronic Engineering, The University of Melbourne, Parkville, Victoria 3010. Australia; 2 School of Community Health, Charles Sturt University, Albury, New South Wales, Australia

## Abstract

**Background:**

Cardiac autonomic neuropathy (CAN) in diabetes has been called a "silent killer", because so few patients realize that they suffer from it, and yet its effect can be lethal. Early sub clinical detection of CAN and intervention are of prime importance for risk stratification in preventing sudden death due to silent myocardial infarction. This study presents the usefulness of heart rate variability (HRV) and complexity analyses from short term ECG recordings as a screening tool for CAN.

**Methods:**

A total of 17 sets of ECG recordings during supine rest were acquired from diabetic subjects with CAN (CAN+) and without CAN (CAN-) and analyzed. Poincaré plot indexes as well as traditional time and frequency, and the sample entropy (SampEn) measure were used for analyzing variability (short and long term) and complexity of HRV respectively.

**Results:**

Reduced (p > 0.05)_Poincaré plot patterns and lower (p < 0.05) SampEn values were found in CAN+ group, which could be a practical diagnostic and prognostic marker. Classification Trees methodology generated a simple decision tree for CAN+ prediction including SampEn and Poincaré plot indexes with a sensitivity reaching 100% and a specificity of 75% (percentage of agreement 88.24%).

**Conclusion:**

Our results demonstrate the potential utility of SampEn (a complexity based estimator) of HRV in identifying asymptomatic CAN.

## Background

As many as 22% of people with type 2 diabetes mellitus (DM) suffer from cardiac autonomic neuropathy (CAN) which leads to impaired regulation of blood pressure, heart rate and heart rate variability (HRV). Around 75% of people with diabetes die from cardiovascular disease such as heart attack and stroke [1]. Silent ischemia is significantly more frequent in patients with than in those without CAN (38% vs 5%) [2]. Early sub clinical detection of CAN for risk stratification and intervention for preventing the potentially serious consequences of CAN especially in people with diabetes are therefore of prime importance.

A noninvasive Ewing test battery [3] specifically designed for identifying CAN consists of five tests but is less sensitive to changes associated with cardiac autonomic neuropathy compared to spectral methods[4]. Combining the five test results allows classification into mild, moderate or severe CAN. The Ewing battery however requires patient cooperation and more importantly is often not able to be performed due to co-morbidities in the patients that would benefit most [5]. These co-morbidities include existing heart or respiratory disease, which is a counter indication for the Valsalva manoeuvre. Use of antihypertensive medication influences the outcome of the lying to standing test that measures blood pressure changes on standing and identifies orthostatic hypotension. The hand grip test is hindered by lack of strength in the elderly and more often by arthritis in the hands. The lying to standing heart rate (HR) test is the easiest test to perform, although it may be difficult for some with a lack of mobility as is often found in the elderly [6].

New methods that are non-invasive and independent of patient cooperation are preferable in the diagnosis of CAN but still require further research to understand their sensitivity and specificity in risk stratification for CAN. The most common method used is heart rate variability analysis [7]. A change in HRV is regarded as one of the early signs of cardiac autonomic neuropathy [8]. However, conventionally used time and frequency domain parameters of HRV are not always suitable for analysis because of the non-stationarity characteristic of the ECG recordings and the presence of nonlinear phenomena in the physiological signal's parameter variability. Only a few studies have applied new parameters based on nonlinear dynamics theory to HRV analysis in DM patients [9,10]. Application of new signal processing techniques based on nonlinear dynamics provides supplementary information (i.e. hidden underlying mechanisms) about systems involved in cardiovascular function and pathology. The visual analysis of variability by the Poincaré plot [11] and quantification of the unpredictability and complexity of the heart rate using sample entropy [12] are increasingly used because they can be computed from shorter HRV records that are used in community screening.

Therefore, the aim of this study was to ascertain how and which of the variability and complexity parameters of the heart rate variability derived from the Poincaré plots and sample entropy are different in DM patients with CAN (CAN+) compared with DM patients without CAN (CAN-) as a first step to determining the utility of these parameters in identification of asymptomatic CAN during supine rest.

## Methods

### Subjects and ECG signals

After standard exclusion criteria were applied to ensure that any changes in HRV detected were due to the severity of the diabetes, seventeen patients with Type 2 diabetes mellitus were included in the study. Nine patients were CAN+, whilst the remaining eight were CAN-, being without clinical signs and symptoms of CAN. The research protocol was approved by Charles Sturt University Ethics in Human Research Committee (03/164). Exclusion criteria included for the CAN-, those with a history of cardiac pathology, hypertension or on antihypertensive medication and those with less than 85% qualified sinus beats. For the CAN+ all participants with greater than 85% qualified sinus beats were included. ECGs were recorded over 20 minutes using a lead II configuration (Maclab ADInstruments Australia) and recorded on Macintosh Chart version 4 with a sampling rate set at 400 Hz and a notch filter at 50 Hz. CAN+ was determined using the reference range for the lying to standing heart rate change (30:15 ratio) reported by Ewing [3]. Table 1 summarises the clinical variables of the patient group.

**Table 1:** Characteristics of the study population

	Age (Years)	Gender (M/F)	BMI (kg.m2)	DAN ratio
CAN+(n = 9)	52 ± 12	5(4)	31.69 ± 5.47	0.95 ± 0.03

CAN-(n = 8)	56 ± 14	3(5)	31.39 ± 4.98	1.10 ± 0.04

ECG signals were edited using the MLS310 HRV module (version 1.0, ADInstruments, Australia) included in the Chart software package. High frequency noise was removed with a 45 Hz low-pass filter and a 3 Hz high pass filter adjusted for wandering baseline. Ectopic beats were selected visually and deleted manually. Linear interpolation was used to replace ectopic beats that occur immediately before and after the ectopic interval. Intervals between successive R waves of the QRS complex (i.e. instantaneous heart rate (IHR) in beats per min (bpm) = 60/R-R intervals in seconds) were calculated using the algorithm developed by Pan and Tomkin [13]. The HRV analysis described in the following sections was performed on IHR of 1000 beats.

### Conventional HRV indices

We quantified several time domain HRV parameters: mean IHR, standard deviation of normal IHR data (SDNN) and the square root of the mean squared difference of the successive IHR data (RMSSD). Spectral analysis was performed on linearly resampled (1 Hz) time series using Welch's method [14]. The 256-point fast Fourier transform was repeatedly computed with 50% overlap between adjacent segments. Then spectral power of each segment was computed and averaged. Hanning window was applied to avoid spectral leakage. Subsequently, spectral powers in the low frequency (LF) band (0.04–0.15 Hz) and high frequency (HF) band (0.15–0.40 Hz) were obtained by integration. The normalized LF and HF powers were calculated by LF/(Total Power-VLF) and LF/(Total Power-VLF) respectively as per Task force recommendation [15]. The power in the very lower frequency (VLF) band was set at ≤ 0.04 Hz.

### Poincaré plot analysis

The Poincaré plot is a popular two-dimensional visualization tool for dynamic systems due to its intuitive display of the dynamic properties of a system from a time series. The length (SD2) and the width (SD1) of the Poincaré plot images represent short and long-term variability of any nonlinear dynamic system [11]. We developed mathematical formulations that relate each measure derived from the Poincaré plot geometry to well-understood existing heart rate variability indexes [11]. A strong correlation was found when comparing high frequency power of heart rate signals (modulated by parasympathetic nervous system) to SD1 [16]. SD2 was found to be well correlated with both low and high frequency power (modulated by both the parasympathetic and sympathetic nervous system) [16]. The Poincaré plot was generated as a scatter plot of current instantaneous heart rate (IHR) against the IHR immediately preceding it. Using the method described by Brennan [11], these plots were used to extract indexes, such as length (SD2) and width (SD1) of the long and short axes of Poincaré plot images.

### Sample entropy analysis

Sample entropy (SampEn) values of IHR signals from all subjects were calculated. SampEn was developed to reduce the bias caused by the self matching in approximate entropy which is a mathematical approach to quantifying the complexity and regularity of a system [12]. SampEn is defined as the logarithmic likelihood that the patterns of the data that are close to each other will remain close for the next comparison within a longer pattern. SampEn does not use a template-wise approach when estimating conditional probabilities. It only requires that one template find a match of length m+1, then it computes the logarithm of a probability associated with the time series as a whole. Mathematical derivation can be found in previous literature [12]. A brief description is as follows.

Given a sequence of total **N **numbers of IHR such as IHR (1), IHR (2),........., IHR (N). To compute SampEn of each IHR data set, m-dimensional vector sequences p_m _(i) were constructed from the IHR series [p_m _(1), p_m _(2),................, p_m _(N-m+1)], where the index i can take values ranging from 1 to N-m+1. If the distance between two vectors p_m _(i) and p_m _(j) is defined as |p_m_(j) - p_m_(i)|, ${\text{C}}_{\text{i}}^{\text{m}}(\text{r})=\frac{1}{\text{N}-\text{m}+1}$ [number of vectors such that |p_m_(j) - p_m_(i)| < r and i ≠ j]

Where **m **specifies the pattern length which is 2 in this study, **r **defines the criterion of similarity which was varied from 10~90% of the standard deviation of IHR data (N = 1000 beats). C_i_^m^(r) is considered as the mean of the fraction of patterns of length *m *that resemble the pattern of the same length that begins at index i. Then the SampEn is computed by using the following equation:

(1) $$\text{SampEn}(\text{N},\text{m},\text{r})=\ln\left[\frac{\sum_{\text{i}=1}^{\text{N}-(\text{m}-1)}{\text{C}}_{\text{i}}^{\text{m}}(\text{r})}{\sum_{\text{i}=1}^{\text{N}-\text{m}}C_{\text{i}}^{\text{m}+1}(\text{r})}\right]$$

We divided the data set into smaller sets of length, i.e., m = 2. The next step is to determine the number of subsets that are within the criterion of similarity r, excluding the self match. Then we repeat the same process for the second subset until each subset is compared with the rest of the data set. This process computes $\sum_{\text{i}=1}^{\text{N}-\text{m}+1}{\text{C}}_{\text{i}}^{\text{m}}(\text{r})$ part of equation (1) and N-m+1 = 1000-2+1 = 999. We then repeated the same process for m = 3. Sample entropy is then calculated using equation (1).

### Surrogate data analysis

To prove any intrinsic relationship of the heart rate control system with SampEn, we followed a method of surrogate data analysis introduced by Theiler *et al. *[17]. For each IHR series of all subjects, 10 surrogate IHR series were obtained by randomly shuffling the original series. Each surrogate data set had an identical IHR distribution (i.e., same mean, SD, and higher moments) as the original data sets and differed only in the sequential ordering of IHR series. SampEn values are computed for 10 surrogate data series. The mean values of the surrogate SampEn were then compared with the SampEn computed for the original IHR data series. It was assumed that the presence of the nonlinear structure in the dynamics of the original data exist, given a statistically significant difference is found when comparing the calculated SampEn of the original series and the mean SampEn of the randomly selected surrogate data series. The number of SD (standard deviations) (i.e. d) between the mean of original indexes and the mean of the indexes of the surrogate data sets (i.e. d = (original – surrogate)/SD of surrogate) was computed. If d > 3, the difference between the original data set and the surrogate data set was considered statistically significant [18].

### Statistical analysis

Results were expressed as means ( ± SD). The non-parametric Mann-Whitney U-test was performed to allow for pairwise testing for significant differences of HRV parameters between the two groups. A value of p < 0.05 was considered significant. The non-parametric test was used because of the non-Gaussian distribution of the variables as ascertained by the Lilliefors test. The relative importance of HRV features was determined by receiver-operating curve (ROC) analysis [19], with the area under the curve for each feature represented by ROC area. A ROC area value of 0.5 indicates that the distributions of the variables are similar in both populations. Conversely, a ROC area value of 1.0 would mean that the distribution of the variables of the two populations do not overlap at all

### Classification

A classification tree was then built using the discriminant variables as indicated by the ROC curves. The first variable used was the one that identified the best separation between the CAN+ and CAN- subjects. The other variables were then introduced according to a descending order of discriminative capacity. For each continuous variable, the cutoff value acting as a separator to make the decision was chosen as that offering the smallest number of misclassification, i.e. that which minimises the sum of false-positive and false-negative results. Sensitivity, specificity and percentage of agreement were calculated on a learning sample of this last analysis. The principle of the Classification and Regression Trees (CART) method [20] is to look at all possible splits for all variables included in the analysis. The results are in the form of an inverted tree. CART begins with a root node and, through a process of yes/no questions, generates descendant nodes. Some nodes are terminal nodes, meaning that a final determination for classification is reached while other nodes continue to be split until terminal nodes are reached. Initially the method CART uses to select its questions for splitting nodes needs to be addressed. Then each splitting rule is ranked in order on the basis of a goodness-of-split criterion. Once a best split is found, CART repeats the search process for each subsequent node, continuing recursively until further splitting is impossible or stopped. Then, CART proceeds by growing trees until it is not possible to grow them any further. It generates a maximal tree and a set of subtrees. A leave-one-out cross-validation scheme was adopted to evaluate the generalization ability of the tree. Cross-validation procedures have been used in a number of classification evaluations, particularly for limited data sets [21]. In this scheme the data set was uniformly divided into 17 subsets with one used for testing and the remaining 16 records used to construct the decision tree. This was repeated for other subsets so that all subsets were used as the testing sample.

## Results

### Conventional HRV parameters

All assessed conventional HRV parameters (except mean IHR) in the time domain (SDNN, RMSSD) as well as frequency domain (LF, HF) were reduced in CAN+ patients (Table 2). However, results are not statistically significant.

**Table 2:** HRV indices of CAN+ and CAN- groups

	CAN+	CAN-	p value	ROCarea
Mean HR (bpm)	69 ± 7	65 ± 8	0.30	0.65

SDNN (bpm)	4.55 ± 1.61	5.62 ± 2.3	0.23	0.65

RMSSD (bpm)	3.43 ± 1.41	4.57 ± 2.7	0.21	0.58

SD1 (bpm)	2.43 ± 1.46	3.23 ± 2.26	0.50	0.61

SD2 (bpm)	5.95 ± 1.50	7.25 ± 2.16	0.33	0.56

SD1/SD2	0.39 ± 0.15	0.42 ± 0.11	0.60	0.70

LF (n.u.)	0.54 ± 0.14	0.61 ± 0.14	0.35	0.61

HF(n.u.)	0.38 ± 0.14	0.45 ± 0.14	0.35	0.61

LF/HF	1.86 ± 0.89	1.63 ± 1.70	0.07	0.68

SampEn (m = 2, r = 0.3*SD)	1.09 ± 0.38*	1.48 ± 0.39	0.04	0.79

Surrogated SampEn	1.73 ± 0.45#	1.96 ± 0.29	0.64	0.55

Mean ± SD of HRV parameters of IHR (N = 1000 beats) of nine CAN+ and eight CAN- subjects. * significant (p < 0.05) difference between CAN+ and CAN-. # The values of d [see methods section for details] were found to be greater than 3 between SampEn and surrogated SampEn values of the two groups.

### Poincaré plot indexes

**Figure 1:**
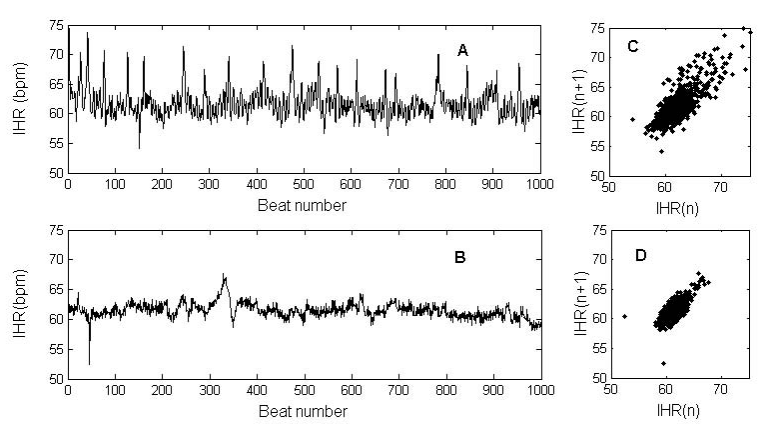
**Poincaré plots**. Top panels show IHR time series from a CAN- subject (A) and the corresponding Poincaré plot (C). Bottom panels show IHR time series from a CAN+ (B) and the corresponding Poincaré plot (D).

Seventeen ECG recordings were analysed. Two representative examples of IHR time series and their corresponding Poincaré plots taken from the HRV patterns of CAN+ and CAN- DM patients are presented in Figure 1. The IHR (Figure 1A) of a CAN- subject with mean IHR of 61 ± 2 bpm, and its corresponding Poincaré plot (Figure 1B) with indexes SD1 = 2.32, SD2 = 6.67, SD1/SD2 = 0.34, are visually different from the IHR characteristics of a CAN+ subject (Figure 1C) with similar mean IHR 61 ± 1 bpm, and its corresponding Poincaré plot (Figure 1D) with indexes SD1 = 1.49, SD2 = 3.24, SD1/SD2 = 0.46. Table 2 summarizes the results from average values of Poincaré indexes of the two groups. Although a marked reduction of SD1 and SD2 in the CAN+ group was observed, no significant differences were found.

The principle of Poincaré plot construction is taken from nonlinear dynamics theory, but indexes used for its quantification are essentially linear [11]. However, this plot can provide supplementary information about beat to beat HRV structure which cannot be obtained by conventional time and frequency domain analysis [22]. From the Poincaré plot indexes, CAN+ subjects had all measures reduced (Table 2). The decreased long term HRV (represented by the length, SD2) and decreased beat to beat HRV (represented by the width, SD1) was expected in CAN+ patients as the reduction of Poincaré plot indexes (SD1, SD2) was confirmed in other studies with parasympathetic nervous system dysfunction [23,24].

SD1/SD2 represents the ratio of short term and long term variability. The smaller SD1/SD2 ratio for the CAN+ subjects reflects that a lower percentage of its overall variance is beat-to-beat variance. However, no significant difference was found between the SD1/SD2 ratio values of the two groups.

### SampEn values of IHR of CAN+ and CAN-

**Figure 2:**
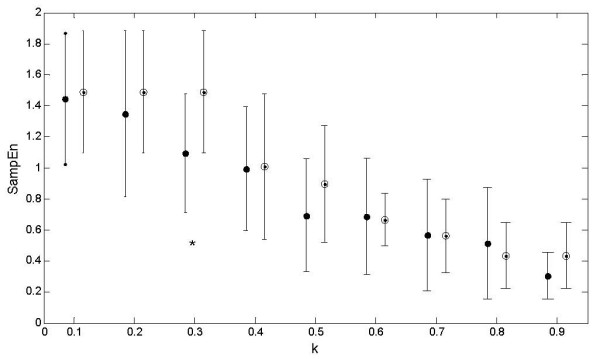
**Error bars**. Mean and standard deviation of SampEn values of IHR signals(N = 1000 beats) of CAN- (blank circle) and CAN+ (black circle) patients over the various r (= k*SD).*means significant (*p *< 0.05) difference. SD = Standard deviation of IHR.

**Figure 3:**
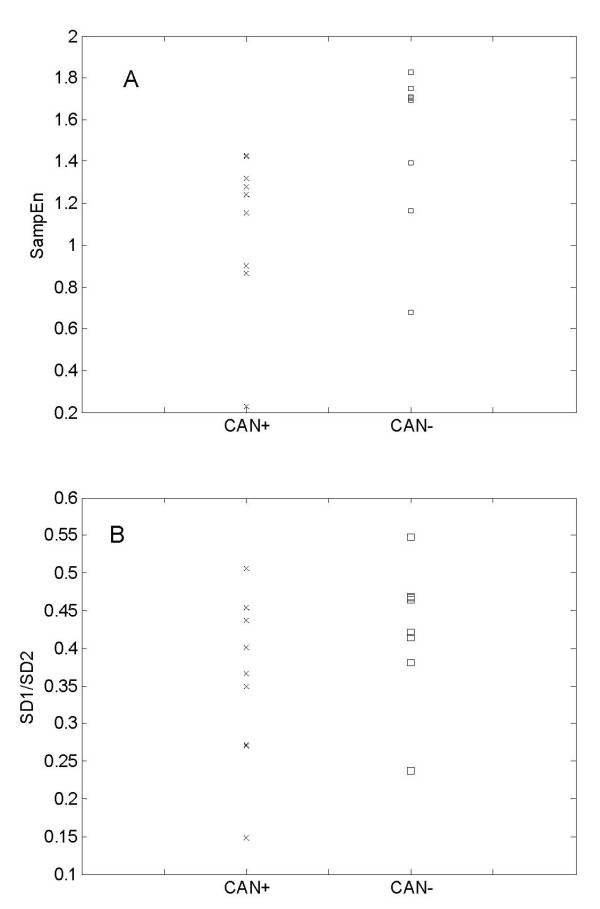
**SampEn values of IHR signals of CAN- and CAN+ patients**. SampEn (m = 2, r = 0.3SD) values (Panel A) and SD1/SD2 values (Panel B) of IHR signals of CAN- and CAN+ patients.

To explore the complexity of the heart rate variability, the sample entropy (SampEn) of the IHR signals was calculated. Figure 2 demonstrates the change of SampEn with **m **= 2 and **r **= 0.1*SD to 0.9*SD of IHR data for CAN+ and CAN- subjects. The mean values of SampEn of the CAN- group were found to be higher than that of CAN+ at all r values except at 0.8*SD. Statistically, CAN+ and CAN- were found to be significantly different (p < 0.05) only at r = 0.3*SD. SampEn values at different r values are summarized in Table 3. Figure 3 shows the SampEn (m = 2, r = 0.3*SD) values of all subjects in this study. Lower values of SampEn reflect more regular time series while higher values are associated with less predictable (more complex) time series. The lower SampEn values for the CAN+ group indicates an increase in regularity and a decrease in complexity in the IHR. A decrease in IHR complexity in CAN+ group indicates the impairment of the cardiovascular control system as found in other studies [10,24]. In order to prove if there are any physiological mechanisms involved, the use of surrogate data analysis was employed to remove the underlying control mechanism (time series structure in beat to beat heart rate) and to increase the degree of randomness. Mean values of the surrogate SampEn of IHR data in the CAN+ group (Table 3) were found to be no more significantly (*p *> 0.05) different to the surrogate SampEn in the CAN- group. Also the mean values of the surrogate SampEn were found to be significantly (d > 3) higher than that of the original SampEn values of IHR data in both groups reflecting the effects of randomness. Therefore, the fluctuations in beat to beat heart rate dynamics are not randomly executed rather they are modulated by a cardiac regulation system which follows a complex pattern in CAN- but changes with neuropathy in CAN+ to a more regular, non-complex pattern.

**Table 3:** Sample entropy values of CAN+ and CAN- groups

	CAN+	CAN-	p values
SampEn (m = 2, r = 0.1SD)	1.44 ± 0.42	1.48 ± 0.39	0.501107

SampEn (m = 2, r = 0.2SD)	1.24 ± 0.53	1.48 ± 0.39	0.437903

SampEn (m = 2, r = 0.3SD)	1.09 ± 0.38	1.48 ± 0.39*	0.045431

SampEn (m = 2, r = 0.4SD)	0.99 ± 0.40	1.01 ± 0.46	0.885458

SampEn (m = 2, r = 0.5SD)	0.69 ± 0.36	0.89 ± 0.37	0.136328

SampEn (m = 2, r = 0.6SD)	0.68 ± 0.37	0.66 ± 0.16	0.259022

SampEn (m = 2, r = 0.7SD)	0.56 ± 0.36	0.56 ± 0.23	0.485993

SampEn (m = 2, r = 0.8SD)	0.51 ± 0.36	0.43 ± 0.21	0.647036

SampEn (m = 2, r = 0.9SD)	0.30 ± 0.14	0.43 ± 0.21	0.319756

Mean ± SD of SampEn (m = 2, N = 1000) values of CAN+ and CAN- groups with r values (0.1~0.9SD). * significant (p < 0.05) difference between CAN+ and CAN-.

### Simple algorithm to identify CAN+

**Figure 4:**
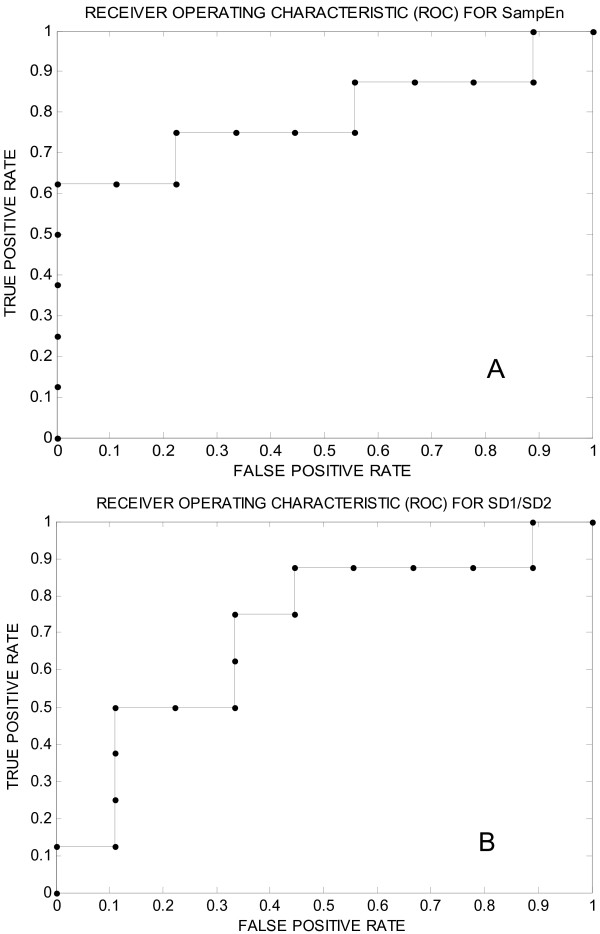
**ROC curves**. ROC (receiver operating characteristics) curves showing true positive (sensitivity) and false positive rate (1-specificity) for various thresholds using sample entropy (SampEn) (Panel A) and SD1/SD2 (Panel B) across 9 CAN+ subjects and 8 CAN- subjects. Areas under ROC curves are 0.79 and 0.70 for SampEn and SD1/SD2 respectively.

**Figure 5:**
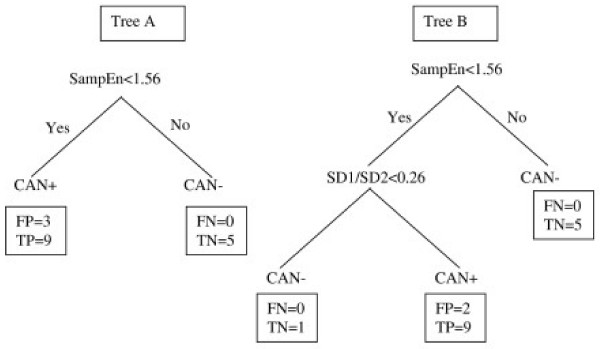
**Tree classifications**. Classification tree illustrating the combination of threshold values of SampEn and SD1/SD2, characterizing CAN+/- in the study. Tree (A) shows accuracy = 82.35%, sensitivity = 100% and specificity = 62.5%. Tree (B) shows accuracy = 88.24%, sensitivity = 100% and specificity = 75.0%. All individual numerical values are indicative of the number of subjects in each terminal node of the tree. TP: true positive, FP: false positive, TN: true negative; FN: false negative.

ROC curves were built separately for each HRV variable (Table 2). SampEn appeared as the best variable for separating the groups (ROC area = 0.79 shown in Figure 4A; p = 0.04) Results of the optimal but simple tree built using CART methodology are presented in Figure 5 (Tree A & B). Tree A uses SampEn only and tree B uses SD1/SD2 values in addition to SampEn. The SampEn represents the most important variable (first node) followed by SD1/SD2. The cut off values for SampEn and SD1/SD2 to decide 'yes' or 'no' were found to be 1.56 and 0.26 respectively. The sensitivity reached was 100% for both trees but the specificity reached 75% when the two variables were used.

## Discussion

Participants in the study were identified as CAN+ by the use of the lying to standing HRV (LS-HRV) test as suggested by Ewing and others to be useful as an indicator of autonomic dysfunction in clinical testing Ewing [5,25]. The use of one test only was based on several disadvantages associated with tests of the 5-test Ewing battery. Thus the Valsalva and sustained handgrip test depend on patient effort and compliance. The postural fall of blood pressure may be unreliable as in people with diabetes it varies throughout the day, being linked to the timing of insulin injections and patients with fluid retention may have extensive autonomic damage but without postural hypotension. Assessment of heart rate associated with deep breathing requires special equipment that indicates depth and cycle of breathing [9]. In addition it has been recommended by diabetologists that the use of lying to standing heart rate change is clinically acceptable [6]. The group of participants analysed are representative of the general cohort attending the clinic expect that they are not hypertensive nor are on antihypertensive or antiarrhythmic medication. This selection was necessary to identify changes in HRV due to diabetes rather than including confounding variables such as antihypertensive medication [26].

Our investigations using time and frequency, Poincaré plot and SampEn of HRV demonstrate the use of variability and complexity analysis in cardiac dynamics in diabetes patients where the complexity of heart rate decreases in patients with cardiac autonomic neuropathy compared to those without. The major finding of this study shows that as compared to conventional HRV indices and Poincaré plot parameters, SampEn of HRV based on the complexity information of heart rate was able to better distinguish diabetic patients with cardiac autonomic neuropathy from the diabetic patient without cardiac autonomic neuropathy.

Although statistically not significant possibly due to small sample size, CAN+ appears to be accompanied by a reduction in heart rate variability indices as measured by SDNN, RMSSD, SD1, SD2 (Table 2). In another study [9], a reduction in HRV was regarded as one of the early signs of cardiac autonomic neuropathy. The characteristic findings of that study in adult diabetic patients with cardiovascular autonomic neuropathy showed resting tachycardia and mostly reduced HRV, which was thought to be the earliest sign of cardiac autonomic dysfunction. In our study we did not find any significant difference in HRV indices between diabetic patients with and without CAN, but we found reduced time and frequency domain HRV parameters in CAN+ patients. These findings are in agreement with a previous study [24], where the non-significant difference in resting mean heart rate was often accompanied by significant differences in conventional HRV parameters in diabetic patients of various ages. These findings indicate possible dysfunction of sympathetic and parasympathetic component of autonomic nervous system in diabetic patients with CAN. Task force [15] proposed standard HRV measures that use heart period signals (RR intervals). In this study, we utilized the same mythologies using heart rate signals (IHR points). It could be argued that since the relationship between IHR and RR intervals is nonlinear, different conclusions on the behavior of HRV can be obtained from the same experiment according to the signal selected. In this study IHR was used because it is simple to understand heart rate rather than RR intervals. Recent research results [27] show that no appreciable difference was observed in the estimation of LF/HF power ratio, LF (normalized) and HF (normalized) power of IHR and RR intervals. This is expected because these indexes refer only to features of the spectral shape but do not take into account the absolute value of the spectral components. In this study, we adopted the normalized spectral measures rather than the absolute values (see Table 2).

The major aim of our study was to identify if complexity information of HRV is able to distinguish cardiac dynamic changes of diabetic patients with CAN from those without CAN. The complexity of HRV patterns as measured by SampEn, was found to be significantly lower in CAN+ subjects [Table 2]. This important association between variability and complexity organization of heart beat fluctuations may be specific for the process of CAN and suggests that there are alterations in the cardiac control mechanism associated with CAN. More specifically, the reduced SampEn of heart rate complexity with CAN suggests a reduced responsiveness of the cardiac control mechanism to external and internal stimuli and thus a reduced strength of feedback interactions.

A complex system is characterized by inherent limitations in the ability to predict the long-term or emergent behavior of the system. Complexity only increases if the non-linear interactions between structural components actually increase [28]. A defining feature of healthy physiological function is adaptability and the capacity to respond to unpredictable stimuli and stresses. Goldberger et al [29] reported that the absence of a characteristic component in pathology inhibits the emergence of highly periodic behaviors (modelocking), which would greatly narrow functional responsiveness as seen in our results for the CAN+ group. Transitions to strongly periodic dynamics are observed in many other pathologies apart from CAN, including Parkinson's disease (tremor), obstructive sleep apnea, sudden cardiac death, epilepsy, and fetal distress syndrome, to name but a few [30]. The appearance of highly ordered dynamics with pathologic states exemplifies the concept of complexity loss in disease.

In contrast to Goldberger's hypothesis, Vaillancourt and Newell [30] noted increased complexity and increased approximate entropy in several disease states, including acromegaly and Cushing's disease, and hypothesized that disease may manifest with increased or decreased complexity, depending on the underlying dimension of the intrinsic dynamic (e.g. oscillating versus fixed point). A rebuttal to the rebuttal (all published concurrently) [31] noted that others accept the fundamental premise that increased and decreased variability occur in disease.

Pincus provided a mechanistic interpretation of lowered approximate entropy values clinically linked to compromised physiology, based on mathematical analysis, yet linked to physiology [32]. Javorka [24,33] observed reduced entropy values of HRV signals in diabetic patients in compared to control group but did not separate CAN+ and CAN- participants. Flynn et al. indicated a lower detrended fluctuation in patients with CAN+ compared to CAN- [34]. The present observations demonstrate that SampEn decreases in CAN+ patients relative to CAN- diabetic subjects. Statistical results show that the choice of r = 0.3 is the optimal choice to use for categorization as parameter tuning in entropy methods is crucial for their performance. By selecting an appropriate value r in the SampEn algorithm, higher discrimination between CAN- and CAN+ can be achieved. We speculate that it allows an optimal tolerance limit for pattern matching maximizing the difference in SampEn values of the two groups. Optimal selection of m and r has been unexplored area. Pincus interpreted the Approximate Entropy (ApEn) of HRV in infants that r values smaller than 0.1SD causes poor conditional probability (C_i_(r)) estimates whereas for r values larger than 0.25 SD, too much detailed system information is lost [35]. Lewis et al [36] suggested for estimating SampEn, which was designed to reduce the bias of ApEn that there were some differences in the optimal m and r combinations for different physiological states. In another study on SampEn of HRV, Lake proposed to calculate SampEn for a range of r and select the value that optimizes an efficiency matrix [37]. As r increases, the practical concern is that the probability of matches or the conditional probability tends toward 1 and SampEn tends to 0 for all, thereby reducing the ability to distinguish any salient feature in the dataset [37]. In this study, as SampEn at r = 0.3SD can better distinguish the CAN+ from CAN-, we speculate that due to higher variability in IHRs of CAN+/- subjects, an r value at 0.3SD can still retain the detailed information in extracting complexity/regularity information of HRV structures in diabetic subjects.

Our results using SampEn and the Poincaré plot also showed three false positive cases. The three cases may be in the early stage of autonomic neuropathy which is under the sensitivity limit of the lying to standing heart rate test employed to classify into CAN+ and CAN- in our investigation. The combination of the two nonlinear measures of HRV, i.e., SD1/SD2 ratio and SampEn show better accuracy in identifying cardiac autonomic neuropathy. SD1/SD2 describes a relationship between microscopic and macroscopic variability. On the other hand, SampEn extracts the complexity information, a nonlinear behaviour. A combination of complexity structure and scale invariant structure of heart rate could be a useful measure of dynamical stability of autonomic nervous system function underlining the possible benefit in cardiac dysregulation diagnosis. This corroborates previous discussion in the literature that nonlinear measures are more sensitive compared to the Ewing battery or individual tests form the Ewing battery.

The range of the SampEn measure needs to be evaluated in non-diabetic control subjects to elucidate the effect of diabetes on heart rate complexity and a deviation from normal, where either an increase or a decrease, should be observed in CAN+/- diabetic subject. Future research will show whether a change in the unpredictability or randomness of beat to beat heart rate dynamics is associated with cardiovascular autonomic neuropathy in diabetes or with diabetes only.

## Conclusion

In conclusion, the results of the investigation indicate dysfunction of the autonomic control of heart rate in DM patients with CAN+. HRV complexity analysis using short term ECG traces could be effective in detecting CAN. Further research on a large sample size is required to further elucidate the findings of this study and effectiveness of HRV complexity analyses for differentiation between mild and severe CAN+ in diabetic patients.

## Competing interests

The authors declare that they have no competing interests.

## Authors' contributions

HFJ recruited subjects, managed data acquisition and participated to drafting of the manuscript. AHK and MP conceived the study, evaluated the data, performed data analyses and wrote the manuscript. All authors read and approved the final manuscript.

## References

[B1] Australian Diabetes Society (ADS). http://www.diabetessociety.com.au/.

[B2] Johnston SC, Easton JD (2003). Are Patients With Acutely Recovered Cerebral Ischemia More Unstable?. Stroke.

[B3] Ewing DJ, Martyn CM, Young RJ, Clarke BF (1985). The value of cardiovascular autonomic function tests: 10 years experience in diabetes. Diabetes Care.

[B4] Pagani M (2000). Heart rate variability and autonomic diabetic neuropathy. Diabetes, Nutrition and Metabolism.

[B5] Ewing DJ, Clarke BF (1982). Diagnosis and management of diabetic autonomic neuropathy. British Medical Journal.

[B6] Ewing DJ, Campbell IW, Murray A, Neilson JM, Clarke BF (1978). Immediate heart-rate response to standing: simple test for autonomic neuropathy in diabetes. British Medical Journal.

[B7] Rollins MD, Jenkins JG, Carson DJ, McGlure BG, Mitchell RH, Imam SZ (1992). Power spectral analysis of the electrocardiogram in diabetic children. Diabetologia.

[B8] Spallone V, Menzinger G (1997). Diagnosis of cardiovascular autonomic neuropathy in diabetes [abstract]. Diabetes.

[B9] Ziegler D (1994). Diabetic cardiovascular autonomic neuropathy: prognosis, diagnosis and treatment. Diabetes Metab Rev.

[B10] Jelinek HF, Pham P, Struzik ZR, Spence I, Munecazu Tacano (2007). Short term ECG recording for the identification of cardiac autonomic neuropathy in people with diabetes mellitus. Proceedings of 19th International Conference on Noise and Fluctuations: 9–14 September 2007; Tokyo, Japan.

[B11] Brennan M, Palaniswami M, Kamen P (2001). Do existing measures of Poincaré' plot geometry reflect nonlinear features of heart rate variability?. IEEE Trans Biomed Eng.

[B12] Richman JS, Moorman JR (2000). Physiological time-series analysis using approximate entropy and sample entropy. Am J Physiol.

[B13] Pan J, Tompkins WJ (1985). Real time QRS detector algorithm. IEEE Trans Biomed Eng.

[B14] Welch PD (1967). The Use of Fast Fourier Transform for the Esimation of Power Spectra: A Method Based on Time Averaging Over Short, Modified Periodograms. IEEE Transactions on Audio and Electroacoustics.

[B15] Task force of the European Society of Cardiology and the North American Society of Pacing and Electrophysiology (1996). Heart rate variability. Standards of measurement, physiological interpretation, and clinical use. Circulation.

[B16] Brennan M, Palaniswami M, Kamen P (2002). Poincaré plot interpretation using a physiological model of HRV based on a network of oscillators. Am J Physiol Heart Circ Physiol.

[B17] Theiler J, Eubank S, Longtin A, Galdrikian B, Doyne F (1992). Testing for nonlinearity in time series: the method of surrogate data. Physica D: Nonlinear Phenomena.

[B18] Bassingthwaighte JB, Liebovitch L, West BJ (1994). Fractal Physiology.

[B19] Hanley JA, McNeil BJ (1983). A method of comparing the areas under receiver operating characteristic curves derived from the same cases. Radiology.

[B20] Breiman L (1993). Classification and Regression Trees.

[B21] Ripley BD (1996). Pattern Recognition and Neural Networks.

[B22] Kamen PW, Tonkin AM (1995). Application of the Poincaré' plot to heart rate variability: a new measure of functional status in heart failure. Aust N Z J Med.

[B23] Kamen PW, Krum H, Tonkin AM (1996). Poincaré' plot of heart rate variability allows quantitative display of parasympathetic nervous activity in humans. Clin Sci.

[B24] Javorka M, Javorkova J, Tonhajzerova I, Calkovska A, Javorka K (2005). Heart rate variability in young patients with diabetes mellitus and healthy subjects explored by Poincaré and sequence plots. Clin Physiol Funct Imaging.

[B25] May O, Arildsen H (2000). Assessing cardiovascular autonomic neuropathy in diabetes mellitus. How many tests to use?. Journal of Diabetes and its Complications.

[B26] Jideh B, Jelinek HF (2004). Identifying diabetic autonomic neuropathy from heart rate variability. Proceedings of The Australian Health and Medical Research Congress: 22–26 November 2004; Sydney, Australia.

[B27] Castiglioni P, Parati G, Omboni S, Rienzo MD, Allan Murray (1994). Influences of the Selection between Heart-Period and Heart-Rate on the Spectral Evaluation of Heart Rhythm Variability During Exercise. Proceedings of Computers in Cardiology: 25–28 September 1994; Bethesda, MD.

[B28] Nehaniv CL, Rhodes JL (2000). The evolution and understanding of biological complexity from an algebraic perspective. Artif Life.

[B29] Goldberger AL (1997). Fractal variability versus pathologic periodicity: complexity loss and stereotypy in disease. Perspect Biol Med.

[B30] Vaillancourt DE, Newell KM (2002). Changing complexity in human behavior and physiology through aging and disease. Neurobiol Aging.

[B31] Vaillancourt DE, Newell KM (2002). Complexity in aging and disease: response to commentaries. Neurobiol Aging.

[B32] Pincus SM (2000). Approximate entropy in cardiology. Herzschr Elektrophys.

[B33] Javorka M, Javorkova J, Tonhajzerova I, Javorka K (2005). Parasympathetic versus sympathetic control of the cardiovascular system in young patients with type 1 diabetes mellitus. Clin Physiol Funct Imag.

[B34] Flynn AC, Jelinek HF, Smith MC (2005). Heart rate variability analysis: a useful assessment tool for diabetes associated cardiac dysfunction in rural and remote areas. Australian Journal of Rural Health.

[B35] Pincus SM, Goldberger AL (1994). Physiological time-series analysis: what does regularity quantify?. Am J Physiol Heart Circ Physiol.

[B36] Lewis MJ, Short AL (2007). Sample entropy of electrocardiographic RR and QT time-series data during rest and exercise. Physiol Meas.

[B37] Lake DE, Richman JS, Griffin MP, Moorman JR (2002). Sample entropy analysis of neonatal heart rate variability. Am J Physiol Regulatory Integrative Comp Physiol.

